# Impacts of a changing climate and adverse weather events on individuals with spinal cord injury: a narrative review

**DOI:** 10.1016/j.joclim.2026.100678

**Published:** 2026-05-06

**Authors:** Imaan Shah, Michelle McLean, Kazi Rahman

**Affiliations:** Faculty of Health Sciences & Medicine, Bond University, Gold Coast, Australia

**Keywords:** Climate change, Extreme weather events, Spinal cord injury, Preparedness, Adaptation, Mobility

## Abstract

**Background:**

Our climate is changing, with heatwaves, wildfires and hurricanes posing increased risks for individuals with a disability such as spinal cord injury (SCI). Despite growing recognition of disability-related climate risk, the specific challenges for individuals with SCI have not been well-documented.

**Objective:**

This narrative review aimed to broadly explore the literature describing the impacts of a changing climate (e.g., rising temperatures, adverse weather, etc.) on individuals with SCI.

**Methods:**

A structured search was conducted across five databases (PubMed, EMBASE, Scopus, Web of Science, CINAHL) and was supplemented by citation tracking to identify additional relevant studies. Twelve articles were analyzed thematically.

**Results:**

Three themes were identified. In terms of adverse weather events, individuals with SCI are *physiologically* (e.g., due to autonomic dysregulation) *and physically* (e.g., unable to evacuate or seek healthcare due to mobility issues) at risk, experiencing *psychological impacts* (e.g., anxiety). There was a *mismatch between their perceived and actual preparedness for these events,* placing themselves further at risk, due in part to climate skepticism and fatalism. The third theme was i*nequity and disability discrimination.* As the research originates largely from the Global North, those living with SCI globally are generally underrepresented or excluded from climate adaptation policy development. SCI is also often considered with other ‘disabilities’ such as blindness and impaired mental capacity, masking climate impacts.

**Conclusion:**

Individuals with SCI experience several physiological, physical and psychological challenges in the face of increasing climate-related events. Addressing these challenges will require targeted, SCI-specific and inclusive strategies in clinical practice, education, and policy development.

## 1 Introduction

Climate change is a significant global health threat, due to rising temperatures and increasing frequency and severity of extreme weather events [1,2]. It is altering seasonal weather patterns, contributing to more frequent, prolonged, and intense heatwaves, with significant health impacts projected to escalate [3]. Additionally, rising sea surface temperatures are projected to intensify hurricanes and increase storm surge and precipitation, posing heightened adverse weather event risks, particularly in coastal regions [4]. Between 2000 and 2019, extreme weather events affected approximately 1.4 billion people globally, with impacts on health, infrastructure and economic systems [5]. The associated global costs of these events are an estimated US$260.8 billion, which translates to about US$143 billion per year [5].

While the impacts of climate change are global, some countries have been particularly affected. The United States, for example, has experienced a range of destructive extreme weather events, including a five-fold increase in summer wildfire activity since the mid-1990s, attributed largely to anthropogenic climate change [6]. Australia too has been affected by weather events over the past decade. Since 2011, there has been an increase in the frequency of severe tropical cyclones along Australia's northeast coast, particularly Category 4 and 5 systems as well as wildfires [7]. Along Australia’s East Coast, there is an increasing frequency and intensity of heatwaves, droughts, and floods [8]. Additionally, low- and middle- income countries such as Bangladesh and Zimbabwe have experienced intensified climate-related impacts, particularly tropical cyclones, droughts, and flooding [9]. The past decade, but particularly the last few years, has been the hottest on record. According to the World Meteorological Organization (WMO), global average surface temperatures in 2024 reached 1.55 °C above pre-industrial levels, the highest on record [10]. In 2024, over 150 climate disasters were recorded globally, including severe floods, heatwaves, and cyclones [11].

These environmental shifts pose significant risks to human health, with some individuals more at risk such as those with a physical or mental disability [[12], [13], [14], [15], [16], [17], [18]]. In July 2019, the United Nations Human Rights Council proclaimed climate change and disability was a human rights concern, with individuals with disability among the most adversely affected in an emergency. They experience disproportionately higher morbidity and mortality, while being among those least able to have access to emergency support [15]. Professional societies such as the International Society of Physical Rehabilitation Medicine (ISPRM) and the Association of Academic Physiatrists’ (AAP) position statements on climate change and physiatry and climate change and disability echo these concerns, urging for action to include disability-focused strategies in rehabilitation programs and policy-making [16,17].

Individuals with spinal cord injury (SCI) are particularly at risk physiologically during climate-related events such as heatwaves, wildfires and flooding. As some may be affected by autonomic dysreflexia, circumstances associated with excessive heat or interruption of power, medical supplies and caregivers can lead to health issues affecting blood pressure regulation, bowel and bladder function, as well as skin and respiratory issues [19,[20], [21], [22]]. Complications may result in seizures, stroke and even death [21].

A SCI presents physical challenges, such as not being able to access essential services during flooding andhave mobility issues during evacuations [21,22]. Affected individuals' reduced mobility heightens their reliance on assistive devices and caregiver support, particularly during power failures. Deteriorating health and not having access to healthcare or being without power will undoubtedly take its toll psychologically, as would property damage or loss and injury, including to family, friends or neighbors [22].

Guided by the overarching broad research question of how a changing climate impacts individuals with SCI, we initially opted to undertake a rapid review. Based on the types and scope of the articles retrieved, a narrative review was considered more appropriate. A narrative review is more flexible than a systematic, scoping or rapid review as it allows researchers to interpret, critique and summarize a range of studies, the analysis of which is dependent on the context in which the review was conducted. Narrative reviews are also useful for exploring under-researched topics and can provide different perspectives and new insights [23].

Below we describe our findings relating to the impacts of a changing climate (e.g., heat, and other weather events) on individuals with SCI to inform future preparedness planning and improve health outcomes for this particularly at-risk group.

## 2 Methods

### 2.1 Search strategy

The search strategy for this review was framed by the following broad research question: *What are the impacts of a changing climate on individuals with a SCI?* In consultation with a librarian, a comprehensive search strategy was developed using combinations of keywords related to SCI and climate-related events such as floods, heat and wildfires. On 18 March 2025, the search was conducted across five databases includingPubMed, EMBASE, Scopus, Web of Science, and CINAHL, with no date limits. Two primary search strings were developed and adapted to suit the syntax requirements of each database. While the core search logic remained consistent across all five databases, slight variations were required based on database-specific indexing. The PubMed string (Box 1) used [MeSH] terms and [Title/Abstract] fields while the EMBASE string (Box 2) included both exploded terms (/exp) and corresponding free-text keywords to ensure broader coverage.

Box 1 **Search string used for PubMed, combining SCI-related terms with those reflecting climate change and extreme weather events.**

("spinal cord injur*"[Title/Abstract] OR "paraplegia"[Title/Abstract] OR "quadriplegia"[Title/Abstract] OR "tetraplegia"[Title/Abstract] OR "Spinal Cord Injuries"[Mesh])

AND

("climate change"[Title/Abstract] OR "extreme weather"[Title/Abstract] OR "heat wave"[Title/Abstract] OR "heatwave*"[Title/Abstract] OR "heat stress*"[Title/Abstract] OR "heat exhaustion*"[Title/Abstract] OR "heat rash*"[Title/Abstract] OR "flood*"[Title/Abstract] OR "hurricane*"[Title/Abstract] OR "wild fire*"[Title/Abstract] OR "wildfire*"[Title/Abstract] OR "bush fire*"[Title/Abstract] OR "bushfire*"[Title/Abstract] OR "drought*"[Title/Abstract] OR "natural disaster*"[Title/Abstract] OR "Climate Change"[Mesh] OR "Natural Disasters"[Mesh] OR "extreme weather*"[Title/Abstract])

Alt-text: Unlabelled box dummy alt text

Box 2 **The EMBASE string used Emtree terms and title/abstract (ti,ab) fields.**

('spinal cord injur*':ti,ab OR 'paraplegia':ti,ab OR 'quadriplegia':ti,ab OR 'tetraplegia':ti,ab OR 'spinal cord injury'/exp OR 'spinal cord injury')

AND

('climate change':ti,ab OR 'extreme weather':ti,ab OR 'heat wave':ti,ab OR 'heatwave*':ti,ab OR 'heat stress*':ti,ab OR 'heat exhaustion*':ti,ab OR 'heat rash*':ti,ab OR 'flood*':ti,ab OR 'hurricane*':ti,ab OR 'wild fire*':ti,ab OR 'wildfire*':ti,ab OR 'bush fire*':ti,ab OR 'bushfire*':ti,ab OR 'drought*':ti,ab OR 'natural disaster*':ti,ab OR 'climate change'/exp OR 'climate change' OR 'natural disaster'/exp OR 'natural disaster' OR 'extreme weather*':ti,ab)

Alt-text: Unlabelled box dummy alt text

**Fig. 1:**
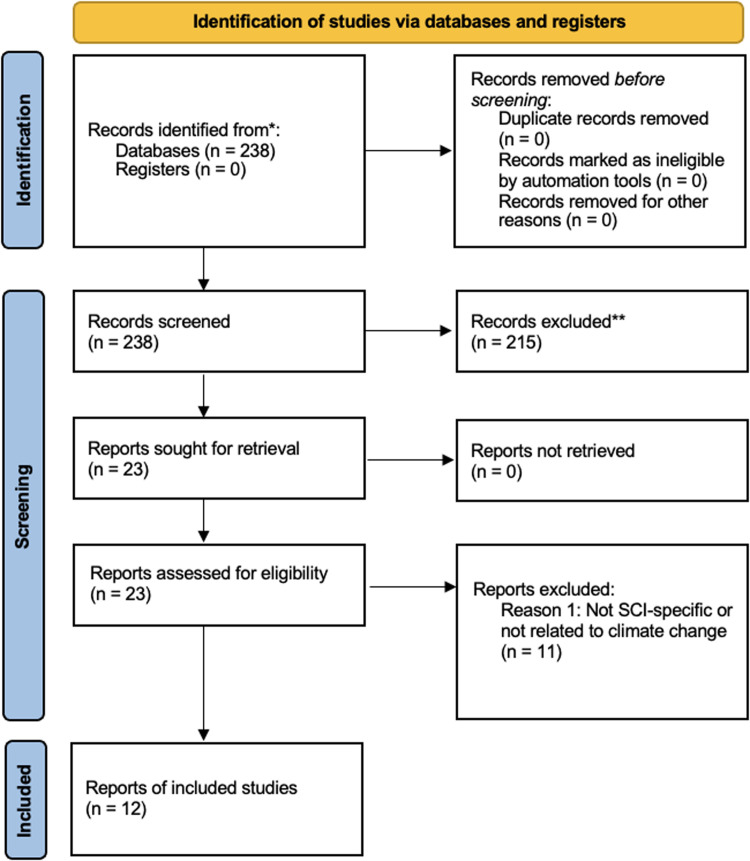
Adapted PRISMA flowchart outlining the number of records identified, screened, excluded, and included in the review process [24].

The PubMed and EMBASE searches yielded 106 and 132 articles, respectively (*n* = 238). Scopus, Web of Science, and CINAHL were also searched using similar terms, but the databases did not yield any additional relevant articles. Fig. 1 provides a summary of the searching, screening and review process further discussed below.

### 2.2 Screening

The 238 articles underwent preliminary screening in terms of the inclusion and exclusion criteria below. Articles were accepted if they:

• Were published in English;
• Included participants with SCI or information relating to individuals with a SCI;
• Explored the effects of climate change, extreme weather events or natural disasters on individuals with SCI;
• Addressed topics such as health impacts, disaster preparedness, access to healthcare, or vulnerability to environmental stressors specific to this at-risk population.

Studies were excluded if they:

• Were not published in English;
• Focused on general disability without any mention of SCI;
• Did not address climate change or related weather events;
• Described earthquakes.

The title and abstract screening were conducted by the lead author (IS), who assessed each article for initial relevance to the overarching question. If deemed potentially relevant, articles were entered into a summary table capturing citation details, study design, population focus, a brief description and a link to the article. Twenty-three articles were selected for screening, 11 from PubMed, 2 from EMBASE, and 10 through forward and backward citation searching of those records. All three authors (IS, KR, and MM) independently reviewed the selected articles using the inclusion and exclusion criteria and then thematically analyzed the content. Further joint critical interrogation of the 23 articles by the research team resulted in 11 articles being excluded, leaving 12 to be included in the narrative review.

## 3 Results

The included articles were published between 2011 and 2024 (Table 1). Six were research articles involving interviews, cross-sectional or convenience surveys. Of the remaining 6 articles, 3 were reviews and 3 were commentaries or perspectives. Eight articles were SCI-specific. The remaining 4 had extractable or applicable SCI content. Seven research or content related to the United States and the Atlantic region, with the remaining 5 classified as international or global (Table 1).

**Table 1:** Details of the 12 articles selected for inclusion in the narrative review.

Authors	Type of article and study	Location	Climate event	SCI-specific or SCI content extracted from general disability
Alexander et al. (2019) [19]	*Research:* Cross-sectional survey of rehabilitation professionals caring for SCI patients	International (Europe, Asia, North America)	Climate change, including extreme weather events	SCI-specific (climate change impacts on clients; education)
Bass et al. (2024) [25]	*Research:* Observational, descriptive, cross-sectional knowledge, attitude and behaviour (KAB) survey of those with SCI, compared with caregivers and the public	USA (Miami)	Extreme heat, flooding and heavy rain	SCI-specific (KAB relating to extreme weather events)
Hogaboom et al. (2013) [26]	*Research:* Descriptive study, convenience sample comparing those with paraplegia and tetraplegia	USA (Pittsburgh)	Natural disasters	SCI-specific (disaster preparedness)
Hogan et al. (2011) [27]	*Research:* Semi-structured interviews with veterans and their veteran health providers	USA	Weather-related natural disasters	SCI-specific (disaster preparedness)
McClure et al. (2020) [28]	*Research:* Convenience sample survey of individuals with SCI using a wheelchair >40h/week with disaster experience	USA	Natural disasters, e.g. floods, hurricanes	SCI-specific (disaster preparedness)
Shapiro et al. (2020a) [21]	*Commentary*	USA and Atlantic coastal regions	Hurricanes	SCI-specific (extreme storm preparation)
Shapiro et al. (2020b) [22]	*Perspective*	USA and Atlantic coastal regions	Hurricanes	SCI-specific (impact of hurricanes; preparedness)
Vasquez et al. (2024) [29]	*Research*: Cross-sectional, self-reporting survey comparing persons with paraplegia, tetraplegia	USA	Raised, warm seasonal temperatures	SCI-specific (high seasonal temperature impacts)
Andreae et al. (2023) [20]	*Narrative review*	Global	Hurricanes	SCI content extracted (SCI risks: Ventilator dependence, thermoregulation, supplies)
Amatya & Khan (2023) [18]	*Review*	Global	Broad climate change phenomena: Heatwaves, floods, droughts, hurricanes, wildfires, air pollution	SCI content extracted (vulnerable group)
Stein & Stein (2021) [30]	*Commentary*	Global	Climate change impacts: Heatwaves, floods, hurricanes, wildfires, air pollution	SCI mentioned (vulnerable group, disability discrimination)
Makuyana & Dube (2024) [31]	*Bibliometric analysis and thematic-scoping review*	Global (South compared with North)	General climate change, extreme weather events	SCI included (human rights and social inclusion issues)

Three themes were identified in the 12 articles, including (1) *physiological, physical, and psychological impacts* of climate or extreme weather events on individuals with SCI (*n* = 7); (2) *preparedness and support systems* during these events (*n* = 8); and, (3) issues relating to health equity, access, and disability discrimination in the context of a changing climate (*n* = 5) (Table 2).

**Table 2:** Thematic synthesis of key findings from included studies (*n =* 12).

Theme	Key findings	Supporting studies (event)
Theme 1: Impacts of climate change, including extreme weather events on individuals with SCI		
**Physiological vulnerability**	Impaired thermoregulation, risk of heat-related illness, fatigue, dehydration, GI, urinary, cardiovascular, respiratory issues (largely due to autonomic dysreflexia), moisture-related skin issues; infections; water and food contamination or shortage; loss of healthy life years	Alexander et al., 2019; Amataya & Khan, 2023; Andreae et al. 2023; Bass et al., 2024; Shapiro et al. 2020a,b; Vasquez et al., 2024
**Physical challenges**	Commute interruptions; interrupted access to healthcare services and medical supplies, especially heat and heavy rain; barriers to evacuation due to assistive technology damage, power failure, infrastructure damage; customized accommodation inaccessible; risk of injury or death; those with a higher level injury more likely to be severely impacted	Amataya & Khan, 2023; Andreae et al., 2023; Bass et al., 2024; Shapiro et al., 2020a,b
**Psychological and emotional distress**	Increased anxiety, major depressive disorder (MDD), PTSD; reduced outdoor activities; social isolation	Alexander et al., 2019; Amatya & Khan, 2023; Andreae et al., 2023; Shapiro et al., 2020a,b
**Theme 2: Disaster preparedness and support systems**		
**Evacuation planning**	Discrepancy between individuals’ perception of preparedness and actual preparedness; reliance on support; difference between home, town or city and work evacuation strategies; individuals with tetraplegia more vulnerable than those with paraplegia; efforts facilitated by communication, teamwork, medical records	Andreae et al., 2023; Hogaboom et al., 2013; Hogan et al., 2011; McClure et al., 2021; Shapiro et al., 2020a,b
**Caregiver support and assistive devices**	Plans depend on caregivers, personal support networks (PSNs); lack of lifting devices increases vulnerability	Andreae et al., 2023; Hogaboom et al., 2013; McClure et al., 2021
**Attitudes and psychological barriers to planning**	Fatalism, past trauma shapes preparedness attitudes (e.g. *“survived Katrina”*), climate denialism, e.g. caregivers less likely to believe climate change is happening than those with a SCI or the public; some with SCI and their caregivers did not consider themselves or their wards more vulnerable to extreme weather; perceptions of vulnerability higher with higher-level SCI	Bass et al., 2024; Hogan et al., 2011
**Disaster preparedness education and training**	Gaps in disaster knowledge among patients and providers; education required for those with SCI, their caregivers and health providers	Alexander et al., 2019; Andreae et al., 2023; Hogaboom et al., 2013; McClure et al., 2020; Shapiro et al., 2020a
**Theme 3: Health equity and disability discrimination**		
**Systemic exclusion from disaster planning and research**	People with disability (including SCI) generally excluded from planning and policy; limited representation in decision-making; disability and climate change scholarship skewed towards mental health and medical sociology	Alexander et al., 2019; Amatya & Khan, 2023; Makuyana & Dube, 2024; Stein & Stein, 2022
**Intersectional vulnerability**	Higher risk for women, minorities, and those living alone. Marginalization, inequity, and human rights issues	Alexander et al., 2019; Amatya & Khan, 2023; Andreae et al., 2023; Makuyana & Dube, 2024; Stein & Stein, 2022

### 3.1 Theme 1: impacts of climate change, including extreme weather, on individuals with SCI

Individuals with SCI face numerous climate-related physiological and physical challenges that also are likely to result in psychological distress.

#### 3.1.1 Physiological vulnerability

The primary physiological impact relates to thermoregulatory dysfunction as a significant physiological vulnerability for individuals with SCI [[18], [19], [20], [21], [22],^30^]. In a US cross-sectional survey, Alexander et al.’s cross-sectional survey of health professionals (HPs) working in SCI care identified those with SCI as one of the most climate-sensitive disability groups due to the inability to maintain a constant core temperature independent of ambient temperature [19]. Health concerns during extreme heat reported by these HPs included difficulty regulating temperature (47.2 %), fatigue (41.6 %), breathing difficulties (40.0 %), and dehydration (37.6 %). The HPs also reported that power outages due to hurricanes can result in secondary complications such as moisture-related skin damage and increased susceptibility to heat- and cold-related illnesses [19].

Although not dealing specifically with climate change, Vasquez and colleagues’ cross-sectional survey of individuals with SCI self-reporting the effects of warmer seasonal temperatures provides a useful lens to examine the impact of higher ambient temperature, an increasing likelihood in current climate modelling, on individuals with different levels of SCI [29]. Individuals with higher-level injuries, i.e., tetraplegia, reported significantly more difficulty cooling down compared with those with paraplegia and non-SCI participants. This impacted daily living activities such as limiting outdoor social activities and attending health appointments. Individuals with SCI, particularly those with higher level injuries (i.e., tetraplegia), experienced more frequent symptoms of heat-induced dizziness and lightheadedness, suggesting that as the level of impairment increases, so does vulnerability to heat-related complications [29].

#### 3.1.2 Physical mobility challenges during disasters

Storm debris, uneven terrain, damaged roads, inaccessible buildings, damaged wheelchairs and power outages were key barriers to mobility and evacuation for individuals with SCI during climate-related disasters [[20], [21], [22],^25^]. Bass et al. [25] highlighted this in their cross-sectional study, in which individuals with SCI reported 1–5 interruptions to daily activity per month due to extreme weather events.

While disruptions such as during heavy rainfall or extreme heat were ‘inconvenient’ and resulted in missed or rescheduled healthcare appointments, other impacts posed more serious threats to health and safety [25]. To this end, Andreae et al. emphasized the vulnerability of individuals with SCI in disaster settings, as restricted mobility impedes activities of daily living (ADLs), including preparing the home, accessing food, and obtaining essential medical supplies and personnel [20]. Participants in Bass and colleagues’ cross-sectional survey reported concerns about water damage to wheelchairs during flood events, as well as loss of mobility due to battery depletion during power outages [25]. Loss of power during extreme weather events presents a critical challenge in terms of safety and mobility for individuals with SCI [[20], [21], [22],^23^]. In particular, elevator failure for individuals living in high-rise buildings puts them at risk of entrapment during hurricanes, with no access to supplies and assistance [22].

Flooding and storm debris further obstruct evacuation, particularly for wheelchair users with limited upper-body strength or propulsion capacity. Damage or destruction of assistive technology and home accessibility features (e.g., ramps and lifts) were common in extreme weather events. Additionally, power outages compromised the charging of mobility devices such as power chairs and scooters, while the loss of indoor lighting creates additional navigation hazards [20,22].

#### 3.1.3 Psychological risks and emotional distress

Several studies have reported psychological vulnerability in the context of climate-related events for individuals with SCI [[19], [20], [21], [22]], including post-traumatic stress disorder (PTSD), major depressive disorder (MDD), and anxiety during hurricanes, largely due to elevated perceptions of threat and reliance on others for evacuation and safety [21,22]. Alexander et al. used the term ‘climate jail’ to describe the prolonged confinement experienced by individuals with SCI during periods of extreme environmental conditions. This isolation was associated with heightened psychological distress, including symptoms of depression, social withdrawal, and a diminished quality of life [19]. Andreae et al. found that individuals with pre-existing trauma or disability-related stressors were more susceptible to storm-related psychiatric symptoms [20].

### 3.2 Theme 2: disaster preparedness and support systems

The literature on evacuation preparedness and support systems for individuals with SCI highlighted gaps in existing evacuation plans, reliance on support persons, and assistive technologies. An individual’s mindset was important in terms of preparedness, with some participants confident in their ability to manage, while others adopted a passive or resigned approach to disaster preparedness.

#### 3.2.1 Evacuation planning

A clear and concerning disconnect between perceived preparedness and actual readiness among individuals with SCI during climate-related emergencies was common [[19], [20], [21],[26], [27], [28]] (Table 2). In their perspective article, Shapiro et al. [22] described how individuals with SCI may be underprepared for emergencies due to lacking essential items such as a working flashlight or sufficient water. This echoes Hogaboom and colleagues’ small US cross-sectional study findings that while some individuals with SCI who were full-time wheelchair users had plans, they mostly overlooked critical components such as identifying designated post-evacuation meeting places, identifying transportation options, having essential supplies, having vital documents on their person or communicating with their personal support networks (PSNs) [26]. Preparedness, measured on a 10-point scale, was generally poor, with a median score of 3/10 for both home and town/city evacuations [26].

McClure et al.’s U.S. survey found that full-time (>40h/week) wheelchair users with SCI often had evacuation plans from their home, but not from the town or city [28]. Interestingly, individuals with tetraplegia were more likely to have an evacuation plan than those with paraplegia, presumably because of their greater need for assistance and therefore a recognition of their vulnerability [28]. Hogaboom and colleagues’ study of full-time wheelchair users, however, found the opposite, i.e., those with tetraplegia had lower median home and town or city evacuation scores than those with paraplegia [26].

Hogan and colleagues’ US semi-structured interview study found substantial variation in how veterans with SCI approached disaster preparedness while living in the community [27]. While some described working closely with caregivers and loved ones to develop tailored plans, others relied on an informal or reactive approach, described by one participant as *“flying by the seat of our pants.”* HPs were found to play a key role in supporting these individuals, often reviewing proposed plans and encouraging adjustments when strategies seemed unrealistic [27]. Preparedness included sending out information packets with contact details for SCI coordinators, planning scenarios (e.g., *“What will you do if your phone doesn’t work?”* or *“Can you manage without electricity for a week?”*), and checklists outlining essential supplies such as medications, medical equipment, and non-perishable food [27]. Healthcare providers in the Veterans Health Administration Spinal Cord Injury and Disorders (VHA SCI/D) system noted that these tools were not only practical, but also served to increase awareness and prompt reflection [27]. Hogan and colleagues did find, however, that preparedness was enhanced by effective communication, teamwork, advanced warning and electronic medical records.

#### 3.2.2 Reliance on caregiver support and assistive devices

There is increased reliance on caregiver support systems and assistive technologies during climate-related emergencies among individuals with SCI [20,26,28]. McClure and colleagues reported that most US wheelchair users’ evacuation plans depended heavily on human assistance [28]. The authors described incidents from Hurricane Katrina in which individuals had died after being unable to connect with their caregivers, underscoring the life-threatening consequences of inadequate planning [28]. Hogaboom et al. found that many individuals who believed they were prepared were missing critical elements in their plans such as Personal Support Networks (PSNs) [26]. PSNs, which include friends, family, neighbors, roommates, and co-workers, play a key role in both pre-emergency planning and aiding during a disaster [26].

Hogaboom et al. recommend establishing a PSN of at least three individuals across locations where individuals with SCI spend considerable time to assist with evacuations. The inclusion of lifting devices such as stair lifts, ceiling lifts, or porch lifts was also emphasized as a vital component of evacuation plans, particularly for individuals who are unable to independently transfer or when members of the PSN were not immediately available [26]. Their study found that 13 of the 21 participants relied on assistive technology devices such as chair lifts, elevators, porch lifts, and ceiling lifts, with significantly greater use among those with tetraplegia (*p* = 0.036) [26].

#### 3.2.3 Attitudes and psychological barriers to planning

Attitudes and psychological factors have been identified as significant barriers to disaster preparedness and evacuation among individuals with SCI [25,27]. Bass’ US-based survey highlights two important insights into attitudes towards vulnerability and climate change. In the first instance, some individuals with SCI did not perceive themselves as being vulnerable, and secondly, only 30 % were convinced that climate change was occurring, which is more than the 12 % of caregivers who were convinced that climate change was happening [25].

Hogan and colleagues’ interviews with US veterans with SCI found that prior traumatic experiences shaped attitudes about disaster preparedness. One veteran expressed a sense of resilience based on their past hurricane experience - *“I’ve survived Katrina… there’s nothing that’s ever going to be that bad again”* [27].

#### 3.2.4 Disaster preparedness education and training for individuals with SCI and for health professionals

Significant gaps in disaster preparedness knowledge and behaviors were identified among individuals with SCI [19,20,28]. To this end, McClure et al. found that only 69.1 % of survey respondents felt confident in their ability to evacuate their city or town during an emergency [28]. The authors recommended targeted education on accessible transportation options and encouraged individuals to proactively engage with local emergency services when standard evacuation services are unavailable [28].

Improving disaster preparedness education among healthcare providers who support individuals with SCI is essential for reducing vulnerability and enhancing emergency response [[19], [20], [21], [22]]. Alexander et al. found that 56 % of healthcare professionals working in SCI care believed climate change had already affected their clients, with 85 % expressing an interest in further education to better support preparedness efforts [19]. Andreae et al. highlighted the vital role of physiatrists (medical doctors specializing in Physical Medicine and Rehabilitation (PM&R)) and other rehabilitation professionals in enhancing patient preparedness by supporting the development of individualized disaster plans, assessing equipment and supply needs, promoting registration for special needs shelters, and encouraging the formation of PSNs [20]. The role of healthcare professionals also extends to post-disaster recovery, including facilitating access to replacement equipment, addressing mental health needs, and helping patients re-establish disrupted care and services [20]. Notably, no studies address disaster preparedness education for informal carers or caregivers, highlighting a significant research gap.

### 3.3 Theme 3: health equity and disability discrimination

People with disabilities, including those with SCI, are often excluded from disaster planning, communication strategies, and climate policy development [18,19,30,31]. This section explores the literature discussing the underrepresentation of individuals with disabilities in climate decision-making and the impact on their access to essential services, critical support, and protection of human rights during extreme weather events (Table 2).

#### 3.3.1 Systemic exclusion from disaster planning and research

People with physical and mental disabilities, including those with SCI, may not be included in disaster preparedness, emergency planning, and communication strategies in the context of a changing climate. Our literature search identified four articles in which authors highlighted marginalization, inequity, and human rights issues. Alexander et al. [19] noted individuals with mobility issues, such as those with SCI, are among the most at-risk groups in climate emergencies, and their absence from preparedness plans raises serious human rights concerns. They also noted that in instances where climate information is available, it is frequently delivered in ways that are not accessible for those with mobility impairments, limiting their ability to prepare or respond effectively [19].

Stein and Stein highlighted the continued exclusion of people with disabilities from climate mitigation and adaptation planning breaches international human rights obligations, particularly those outlined in the Convention on the Rights of Persons with Disabilities (CRPD) [30]. They reported that access to essential services such as early warning systems, transportation, emergency shelters, and evacuation support is often restricted, due to inaccessible information, lack of inclusive planning, and discriminatory attitudes, placing people with disabilities at greater risk during climate-related events [30]. They also emphasized the need for full participation of Disabled Persons’ Organizations (DPOs) in international spaces like the United Nations Climate Change Conference of the Parties (COP). They called for disability rights to be built into both national and global climate policies as a matter of urgency. In addition, they recommended more individuals with disabilities entering the health professions to collaborate in development and disaster risk reduction to address disability discrimination.

Amatya and Khan’s review highlighted that individuals with disabilities, including those with SCI, tend to be excluded from both climate adaptation and mitigation strategies, with limited involvement in planning or decision-making processes [18]. They argue that disability must be embedded at every level of climate planning, from design through to implementation. They emphasize that inclusion must go beyond tokenism, with a focus on leadership and lived experience informing policy [18]. In the final article, Makuyana and Dube point out that disability remains largely overlooked in climate scholarship [31]. Their bibliometric analysis found that most climate-disability research originates from the Global North, with limited attention to disability-specific data or distinct subgroups, particularly individuals with mobility impairments, as well as those who are blind, deaf, or autistic [31]. Research is currently skewed towards mental disability and medical sociology. The authors advocate for a rights-based, participatory approach to climate policy, one that places disability at the center of governance efforts. Despite the Convention on the Rights of Persons with Disabilities (CRPD), these protections are rarely implemented in practical disaster planning [18]. They further point out that many climate policies still lack clear definitions of disability and fail to engage disabled communities in co-creating solutions. This ongoing exclusion, they contend, weakens both the legitimacy and the effectiveness of climate responses, particularly for marginalized groups who are already disproportionately affected [31].

#### 3.3.2 Intersectional vulnerability

Intersectional vulnerability describes how overlapping factors, including gender, race, income, and social isolation, compound disadvantage during climate events [18,20,32]. Andreae et al. described highly vulnerable individuals with disability in relation to hurricanes, such as those living in flood-prone areas, the elderly, pregnant women, Black and Native Americans. They also raised the issue of climate gentrification, i.e., property in low-risk areas become more desirable, resulting in higher prices. While there is no research on the effects of this on individuals with disabilities, this disproportionately affects individuals who are Black, unemployed, and/or renters [20].

In their review, Amatya and Khan highlighted that non-White women living alone or in poverty often face heightened risks during climate events due to barriers in accessing care, timely information, and transportation [18]. Similarly, Stein and Stein argue that when disability intersects with other forms of disadvantage such as racial or economic inequality, barriers to preparedness and safety are exacerbated [32].

Additionally, McClure et al. found that among individuals with SCI, women, older people, those from minority backgrounds, and individuals living alone reported significantly lower levels of preparedness and reduced ability to evacuate. This adds an additional layer of vulnerability to an already at-risk demographic [28]. No studies in our search specifically addressed the experiences of those from culturally and linguistically diverse backgrounds (CALD), highlighting a potential research gap.

## 4 Discussion

### 4.1 Key findings

This narrative review examined the physiological, physiological, and psychological impacts, preparedness barriers, and health equity challenges faced by individuals with SCI during climate-related extreme weather events and disasters. It is important to note that, due to the limited and Global North nature of available research, many of the included studies referred to ‘disability’ broadly (i.e., sight and hearing loss, cognitive, etc.). Notwithstanding, the findings remained highly relevant to individuals with SCI, particularly where studies focused on physical or mobility impairments. Across the included studies, those with SCI experience considerable physiological vulnerabilities, particularly those with tetraplegia. Most notably, impaired thermoregulation during heat events and during power outages increased the risk of heat-related complications, while impacting access to health services [19,21,25,30]. Mobility-related barriers during emergencies, such as inaccessible infrastructure and damaged assistive devices, further hindered evacuation and safety [20,21,26,28]. Mental health challenges, including heightened rates of depression, anxiety, and post-traumatic stress disorder, were also frequently reported [20,22,25]. Regarding preparedness, individuals with SCI lacked comprehensive evacuation plans and relied heavily on caregivers or informal support systems, often without structured coordination [[20], [21], [22],^26^,^28^]. Attitudes such as fatalism or overconfidence, shaped by previous disaster experiences, and climate skepticism, also limited engagement in proactive planning [27].

Finally, this review identified systemic exclusion from climate planning and communication, a lack of disability-inclusive governance, and the critical importance of targeted education for both healthcare providers and individuals with SCI to improve disaster readiness and health outcomes [18,19,30,31].

### 4.2 Interpretation and implications

The literature highlights the need for meaningful inclusion of individuals with SCI and broader disability groups in climate adaptation and disaster planning. Stein and Stein emphasized that emergency systems must be both physically and communicatively accessible [30]. This includes accessible early warning systems, transportation, shelters, and continuity of care during extreme events [30]. Healthcare providers, particularly those in rehabilitation and primary care, play a critical role in recognizing SCI-specific vulnerabilities, such as impaired thermoregulation and dependence on power-based equipment, and in supporting both individuals with SCI and their caregivers in disaster preparedness.

While previous studies have examined disability and climate vulnerability more broadly, this narrative review highlights the specific risks and barriers faced by individuals with SCI, a population with unique physiological, physical, and systemic needs, including continuity of care, reliance on support services, and access to health and social services during emergencies. The findings reinforce that people with SCI face elevated health risks during extreme weather events, not only due to impaired thermoregulation and mobility limitations, but also because of their reliance on assistive devices and caregiver support, particularly if they have tetraplegia. Preparedness planning for this group should include education about emergency water, food and medical supplies, transport needs, registration for special needs shelters, and the establishment of PSNs [21,28].

The review also highlights the striking absence of SCI-focused planning in mainstream disaster frameworks. Despite clear evidence of vulnerability, individuals with SCI (as with other disabilities) remain underrepresented in preparedness policies, education initiatives, and governance structures [18,19,30,31].

By centering individuals with SCI in the context of climate disaster preparedness, this review contributes to a growing call for targeted, rights-based approaches that aim to address gaps in policy, healthcare provider education, SCI-specific public health messaging, and tailored emergency preparedness interventions. Individuals with SCI also require access to targeted preparedness education and scenario-based planning tools that reflect their specific needs and limitations. Resources should be designed to be accessible, actionable, and adaptable across a range of emergencies.

At the policy level, governments and emergency management agencies must integrate SCI-specific considerations into broader climate adaptation strategies, including in low- and middle-income countries. This includes disability-inclusive early warning systems, accessible infrastructure, and the direct involvement of individuals with SCI and advocacy groups in disaster policy development.

### 4.3 Research gaps and future directions

This narrative review identified several critical gaps in the existing literature on the intersection of SCI and climate-related extreme weather events. Most included studies were cross-sectional and self-reported, limiting insights into long-term outcomes and causality. Longitudinal and intervention-based research on preparedness behavior, health outcomes, and disaster recovery is especially limited for this population. Moreover, most of the included studies were conducted in the Global North and high-income countries. This reveals a major research gap, particularly for the Global South, where countries face a greater burden from climate change despite contributing the least to it. Limited resources and weaker infrastructure in these regions increase the vulnerability of individuals with SCI. There is a pressing need for SCI-specific research in low- and middle-income countries, where climate-related events may worsen existing inequalities in healthcare, infrastructure, and emergency preparedness.

It is integral for caregivers to be considered in future research and policies, as they play a key role in supporting and transporting individuals with SCI in the event of climate-related emergencies. Due to this critical role, appropriate training and education are required to ensure they are adequately prepared to respond and assist individuals with complex needs. There is also a need to address climate skepticism, which was found for both those with SCI and caregivers [19,25]. In their 2025 scoping review, Newman and colleagues identified research that indicated that while most PM&R physicians believed climate change was a concern, physicians outside of North America were more likely to believe that climate change would affect patients’ health [32].

Furthermore, studies that focus specifically on individuals with SCI remain scarce. Several publications either grouped SCI with broader disability categories or failed to disaggregate their findings, making it difficult to extract SCI-specific insights [12,18,26,30]. In relation to those with SCI, research generally does not explore any potential differences between risks and impacts in relation to the level of injury. Only two of the SCI-specific research studies reported differential impacts between individuals with paraplegia and those with tetraplegia [26,29], which could potentially be life-threatening as the impacts may be under-estimated. Furthermore, there is also limited representation of marginalized subgroups within the SCI population, including women, individuals from rural or low-resource settings, and minority groups such as migrants and Indigenous people. Future research should be SCI-specific and co-designed with individuals and their carers with lived experience to improve relevance and equity.

### 4.4 Limitations

The search strategy was limited to five databases with only two databases (PubMed and EMBASE) yielding relevant articles. As such, relevant studies indexed in other databases or gray literature may have been missed. The inclusion criteria also limited the review to studies published in English, further narrowing the evidence base and likely excluding research from non-English-speaking regions. Finally, most included studies were conducted in the Global North, in high-income countries, particularly in the US. This limits the applicability of findings to the Global South, to low-and middle-income countries, where climate-related disasters may further exacerbate healthcare inequities, infrastructure gaps, and unmet accessibility needs.

## 5 Conclusions

As extreme weather events become more frequent and severe due to climate change, there is an urgent need for targeted and inclusive preparedness strategies to support individuals with SCI. This review highlights key physiological, physical, and psychological vulnerabilities, gaps in disaster planning, and systemic exclusion from climate adaptation policy affecting this at-risk population.

With a 2023 United Nations Office for Disaster Risk Reduction (UNDRR) global survey finding that only 26 % of the persons with disability say they would be capable of evacuating immediately, and with 84 % not having a personal preparedness plan [33], Chapman and colleagues’ reporting that during disaster events in Australia, individuals with disability were up to four times more likely to be injured or die compared with those without disability, is not surprising [34].

Addressing these issues will require coordinated efforts across clinical practice [16,17], public health education, policy reform, and community involvement to ensure that individuals with SCI are not left behind in disaster response and recovery efforts. The 2015 Sendai Framework for Disaster Risk Management has already established the importance of engaging and partnering with marginalized populations to protect human rights [35] and has been used in the Australian Government’s National Action Plan for Disaster Risk Reduction [36]. In the Government’s recently released National Adaptation Plan (September 2025), based on extensive stakeholder consultation, including the disability sector, those with disability have been recognized as a ‘most impacted’ group who are currently moderately impacted but risk being severely impacted by 2050: *“Climate change and the increasing intensity and frequency of disasters may disproportionately impact people living with disability, who experience high vulnerability during disasters due to physical, informational and systemic barriers.”* (p. 40) [37].

## CRediT authorship contribution statement

**Imaan Shah:** Data curation, Methodology, Writing – original draft, Writing – review & editing. **Michelle McLean:** Writing – review & editing, Supervision, Project administration, Methodology, Formal analysis, Data curation, Conceptualization. **Kazi Rahman:** Writing – review & editing, Supervision, Methodology, Formal analysis, Data curation, Conceptualization.

## Declaration of competing interest

The authors declare that they have no conflict of interest.
